# Na^+^/K^+^-ATPase Alpha 2 Isoform Elicits Rac1-Dependent Oxidative Stress and TLR4-Induced Inflammation in the Hypothalamic Paraventricular Nucleus in High Salt-Induced Hypertension

**DOI:** 10.3390/antiox11020288

**Published:** 2022-01-31

**Authors:** Qing Su, Xiao-Jing Yu, Xiao-Min Wang, Bo Peng, Juan Bai, Hong-Bao Li, Ying Li, Wen-Jie Xia, Li-Yan Fu, Kai-Li Liu, Jin-Jun Liu, Yu-Ming Kang

**Affiliations:** 1 Key Laboratory of Environment and Genes Related to Diseases of Education Ministry of China, Shaanxi Engineering and Research Center of Vaccine, Department of Physiology and Pathophysiology, School of Basic Medical Sciences, Xi’an Jiaotong University, Xi’an 710061, China; qingsuxjd2018@xjtu.edu.cn (Q.S.); 312683766@stu.xjtu.edu.cn (X.-M.W.); lhb15891752503@mail.xjtu.edu.cn (H.-B.L.); lyinxian777@mail.xjtu.edu.cn (Y.L.); xia_wj@stu.xjtu.edu.cn (W.-J.X.); fly4525302@stu.xjtu.edu.cn (L.-Y.F.); liukaili1115@stu.xjtu.edu.cn (K.-L.L.); jupet.1128@xjtu.edu.cn (J.-J.L.); 2 School of Clinical Medicine, Xi’an Jiaotong University, Xi’an 710061, China; a2010031165@stu.xjtu.edu.cn; 3 Department of Anesthesiology & Center for Brain Science, The First Affiliated Hospital of Xi’an Jiaotong University, Xi’an 710061, China; juanbai@mail.xjtu.edu.cn

**Keywords:** alpha 2 Na^+^/K^+^-ATPase, oxidative stress, inflammation, hypothalamic paraventricular nucleus, salt-induced hypertension

## Abstract

Numerous studies have indicated that a high salt diet inhibits brain Na^+^/K^+^-ATPase (NKA) activity, and affects oxidative stress and inflammation in the paraventricular nucleus (PVN). Furthermore, Na^+^/K^+^-ATPase alpha 2-isoform (NKA α2) may be a target in the brain, taking part in the development of salt-dependent hypertension. Therefore, we hypothesized that NKA α2 regulates oxidative stress and inflammation in the PVN in the context of salt-induced hypertension. Part I: We assessed NKA subunits (NKA α1, NKA α2, and NKA α3), Na^+^/K^+^-ATPase activity, oxidative stress, and inflammation in a high salt group (8% NaCl) and normal salt group (0.3% NaCl). Part II: NKA α2 short hairpin RNA (shRNA) was bilaterally microinjected into the PVN of salt-induced hypertensive rats to knockdown NKA α2, and we explored whether NKA α2 regulates downstream signaling pathways related to protein kinase C γ (PKC γ)-dependent oxidative stress and toll-like receptor 4 (TLR4)-induced inflammation in the PVN to promote the development of hypertension. High salt diet increased NKA α1 and NKA α2 protein expression in the PVN but had no effect on NKA α3 compared to the normal salt diet. Na^+^/K^+^-ATPase activity and ADP/ATP ratio was lower, but NAD(P)H activity and NF-κB activity in the PVN were higher after a high salt diet. Bilateral PVN microinjection of NKA α2 shRNA not only improved Na^+^/K^+^-ATPase activity and ADP/ATP ratio but also suppressed PKC γ-dependent oxidative stress and TLR4-dependent inflammation in the PVN, thus decreasing sympathetic activity in rats with salt-induced hypertension. NKA α2 in the PVN elicits PKC γ/Rac1/NAD (P)H-dependent oxidative stress and TLR4/MyD88/NF-κB-induced inflammation in the PVN, thus increasing MAP and sympathetic activity during the development of salt-induced hypertension.

## 1. Introduction

High salt intake is a major determinant for inducing hypertension and cardiovascular diseases (CVDs). Mordecai and Leenen (2012) found that subjects of excessive salt intake had an increased level of Na^+^ in their plasma and cerebrospinal fluid (CSF) [1], which caused the secretion of an ouabain-like compound (OLC) by the hypothalamus and the adrenals, and regulates sympathetic activity and cardiovascular responses [1,2,3,4]. OLC is also considered as a putative ligand of the inhibitory binding site of membrane Na^+^/K^+^-ATPase (NKA) [5]. Additionally, Na^+^/K^+^-ATPase is a vital enzyme that regulates cardiovascular function, neurotransmitters release, sympathetic activity, oxidative responses, and cytokines balance, and it also contributes to the maintenance of cell volume by regulating the resting membrane potential, which distributes in the heart, blood vessels, central nervous system, and renal tubular epithelial cells [6,7,8,9,10,11]. Many researchers indicated that Na^+^/K^+^-ATPase is a ubiquitous membrane-bound enzymes that comprises α (α1–α4) subunits and three β (β1–β2) subunits, transports two Na^+^ ions out of the cell and three K^+^ ions into the intracellular space using energy produced by the hydrolysis of ATP to ADP [12]. The α subunits primarily contain sodium-, potassium-, and ATP-binding sites and exhibit catalytic properties; however, the β subunits are glycosylated proteins that contribute to enzyme maturation and cation affinity. Na^+^/K^+^-ATPase alpha 1 isoform (NKA α1) exists in nearly all tissues; Na^+^/K^+^-ATPase alpha 2 isoform (NKA α2) is the predominant isoform in nervous tissue, skeletal muscle, and the heart [13,14,15]. Na^+^/K^+^-ATPase alpha 3 isoform (NKA α3) is primarily found in the brain, including the hypothalamus [12,16], and Na^+^/K^+^-ATPase alpha 4 isoform (NKA α4) is only expressed in the testis [17,18]. Huysse (2009) showed that NKA α2 in the brain may be a target for the brain OLC during increases in CSF Na^+^ in salt-dependent hypertension [19]. Therefore, we want to know the effect of NKA α2 in the brain on salt-induced hypertension.

Additionally, long-term salt intake elevates reactive oxygen species (ROS) accumulation in the neurons, blood vessels, and kidneys, which takes part in the metabolic disorders and cardiovascular diseases process. Previous studies have also found that a high salt diet exacerbates oxidative stress and the inflammatory responses in the paraventricular nucleus (PVN) of the hypothalamus, an important area for the regulation of cardiovascular activity and blood pressure [20,21,22,23,24,25]. Ras-related C3 botulinum toxin substrate 1 (Rac1) is a vital NAD(P)H subunit that promotes ROS production. Siman et al. (2010) observed that inhibition of Na^+^/K^+^-ATPase activates the renin–angiotensin system (RAS) and augments arterial blood pressure in spontaneous hypertensive rats (SHRs) [4]. We found that RAS also acts on the oxidative stress via the AT1R/PKC γ/Rac1 signaling pathway in salt-induced hypertension [26]. Thus, we hypothesized that NKA α2 in the PVN induces oxidative stress probably by the PKC γ/Rac1-dependent NAD(P)H signaling pathway. In addition, Li et al. (2016) reported that inflammation in the PVN contributes to hypertensive responses via the toll-like receptor 4 (TLR4)/myeloid differentiation protein-88 (MyD88)/Nuclear factor-κB (NF-κB) signaling pathway [27]. Furthermore, NF-κB, a transcription factor, is one of the most important molecules linking chronic inflammation. Thus, we were also interested in whether NKA α2 in the PVN regulates the level of cytokines via the TLR4 signaling pathway. In conclusion, the aim of this study is to examine whether NKA α2 in the PVN activates Rac1-dependent oxidative stress and TLR4-induced inflammation in salt-induced hypertensive rats.

## 2. Materials and Methods

### 2.1. Animals

Male Sprague Dawley rats weighing 150 g to 200 g were purchased from the Experimental Animal Center of Xi’an Jiaotong University. All animals were housed in a temperate (23 ± 2 °C) and light-controlled (12 h light/dark cycle) environment and were provided ad libitum access to rat chow. The study was approved by the Animal Care and Use Committee of Xi’an Jiaotong University (No. xjtu2018-404). The experimental protocols conformed to the Guide for the Care and Use of Laboratory Animals published by the United States National Institutes of Health (US National Institutes of Health Publication No. 85-23, revised 1996).

### 2.2. General Experimental Protocol

**Figure 1:**
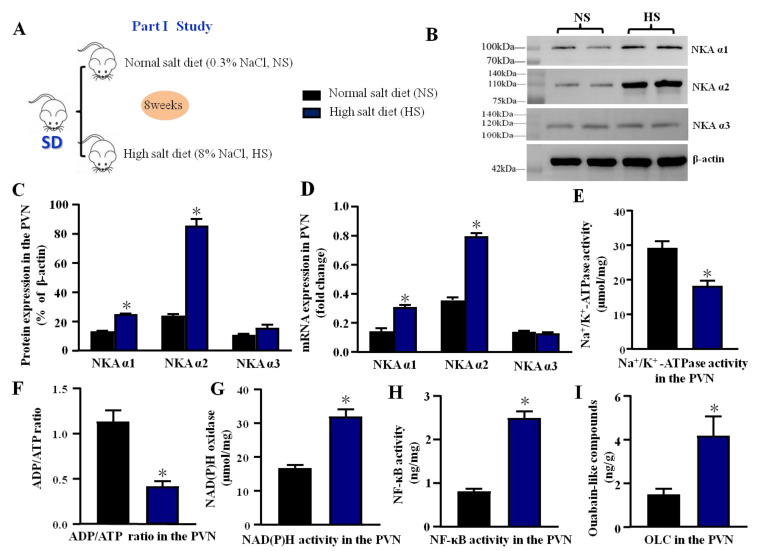
Effect of high salt diet on NKA subunits, Na^+^/K^+^-ATPase, oxidative stress, and inflammation in the PVN (**A**) The flow charts for Part I study. (**B**) The representative immunoblot of NKA subunits (α1, α2, and α3) in the PVN between high salt group (HS) and normal salt group (NS). (**C**) Densitometric analysis of protein expression. (**D**) Bar graph comparing the fold change of mRNA. (**E**) The bar change of Na^+^/K^+^-ATPase activity. (**F**) ADP/ATP ratio. (**G**) NAD(P)H activity. (**H**) NF-κB activity. (**I**) Ouabain-like compounds (OLC) level in the PVN. Values are expressed as the mean ± SEM (*n* = 4–5). * *p <* 0.05 versus control group.

**Figure 2:**
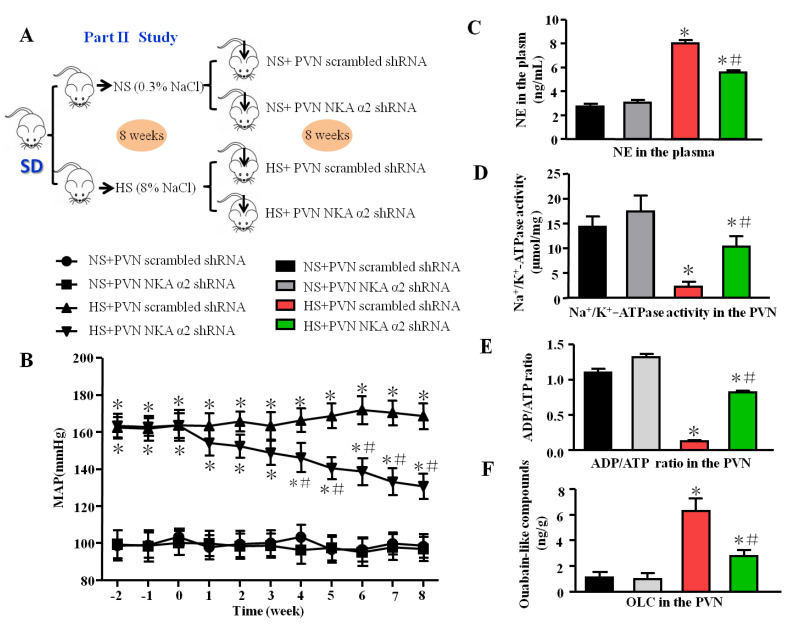
Effect of decreased NKA α2 in the PVN on mean arterial blood pressure, sympathetic activity, NKA, and ADP/ATP ratio in salt-induced hypertension. (**A**) The flow charts for Part II study. (**B**) The line chart of MAP in different groups. (**C**) The histogram of noradrenaline (NE) in the plasma. (**D**) Na^+^/K^+^-ATPase activity in the PVN. (**E**) ADP/ATP ratio. (**F**) Ouabain-like compounds (OLC) level in the PVN in different groups. * *p <* 0.05 versus the normal salt groups (the normal salt + NKA α2 shRNA or normal salt + scrambled shRNA group); # *p <* 0.05, the high salt + NKA α2 shRNA group versus the high salt + scrambled shRNA group. Values are expressed as the mean ± SEM (*n* = 6).

The experimental design is shown in Figure 1A and Figure 2A.

#### 2.2.1. Part I: The Effect of High Salt Diet on NKA Subunits, Na^+^/K^+^-ATPase, Oxidative Stress, and Inflammation in the PVN

Male SD rats (200–230 g) were fed a normal salt diet (0.3% NaCl) or high salt diet (8% NaCl). After eight weeks, all rats were intraperitoneally anesthetized with ketamine (90 mg/kg) and xylazine (10 mg/kg) (i.p.). Microdissection procedures were used to isolate the PVN tissue, which was collected from both sides of the PVN of individual rats and stored at −80 °C for molecular analyses. Moreover, the protein expression and mRNA levels of NKA subunits (α1, α2, and α3) in the high salt group and control group were measured by Western blotting and real-time PCR. ELISA was used to measure the Na^+^/K^+^-ATPase activity, ADP/ATP ratio, ouabain-like compound (OLC), NAD(P)H activity, and NF-κB activity in the PVN.

#### 2.2.2. Part II: The Effect of NKA α2 in the PVN on Rac1-Dependent Oxidative Stress and TLR4-Induced Inflammation in Salt-Induced Hypertension

Male SD rats (200–230 g) were fed a normal salt diet (0.3% NaCl) or high salt diet (8% NaCl). Then, rats in each group underwent bilateral microinjection of NKA α2 short hairpin RNA (shRNA) or scrambled shRNA into the PVN (Figure 1A). The groups were separated as follows: normal salt (NS) + PVN scrambled shRNA, normal salt (NS) + PVN NKA α2 shRNA, high salt (HS) + PVN scrambled shRNA, and high salt (HS) + PVN NKA α2 shRNA. Eight weeks later, some of the rats were perfused with paraformaldehyde, while fresh PVN tissues and plasma were collected from the other rats. ELISA was employed to measure the level of noradrenaline (NE) in the plasma, NKA activity, the ADP/ATP ratio, and ouabain-like compound (OLC) in the PVN.

To assess Rac1-dependent oxidative stress, the protein levels of NKA α2, PKC γ, and phosphorylated Rac1 (p-Rac1) in the PVN were measured by western blotting. The levels of IL-1β, MCP-1, IL-6, TNF-α, IL-4, IL-8, IL-10, and NF-κB activity were measured by ELISA.

### 2.3. NKA α2 shRNA Adenovirus-Associated Virus Preparation

NKA α2 short hairpin RNA (shRNA) oligonucleotides and the scrambled shRNA were provided by Hanbio Biotechnology Co. Ltd. (Shanghai, China). The titer of the adenovirus-associated virus (AAV) is 1 × 10^12^ vg/mL and the serotype is AAV 9. After we obtained the adenovirus-associated virus, the AAV was subpackaged (200 μL/tube) and stored at −80 °C. Before bilateral paraventricular nucleus microinjection, the vectors should be dissolved by putting them on ice.

### 2.4. Bilateral PVN Microinjection of Adenovirus-Associated Virus

In part II, we administered adenovirus-associated virus (AAV) knockdown NKA α2 shRNA or scrambled shRNA via microinjection for 8 weeks. The rats were anesthetized by intraperitoneal injection of ketamine (90 mg/kg) and xylazine (10 mg/kg), and their heads were placed in a stereotaxic apparatus. A skin incision was made, and holes were drilled in the skull at the following stereotaxic coordinates: 1.8 mm posterior to the bregma and 8.5 mm ventral from the skull surface. Scrambled shRNA or NKA α2 shRNA (0.5 μL) was microinjected into the bilateral PVN using a microsyringe (Legato 130, RWD Life Science Co., Ltd., Shenzhen, China) within 10 min once. Animals were administered buprenorphine (0.01 mg/kg) immediately following surgery and 12 h postoperatively.

### 2.5. Measurement of Mean Arterial Blood Pressure

Arterial pressure was measured noninvasively via tail-cuff instrument using a recording system. The procedure for taking the mean arterial blood pressure has been described previously [28,29]. Briefly, unanesthetized rats were warmed to an ambient temperature of 30 °C by placing them in a holding device mounted on a thermostatically controlled warming plate. The rats were allowed to acclimate to the cuff for 10 min before blood pressure measurement. Then, the tail was threaded through the VPR sensor cuff and placed within 2 cm of the occlusion cuff. The body temperature of the animals was kept between 32 °C and 35 °C. When the software showed a stable jagged wave, the blood pressure was measured. The researchers underwent software and animal handling training before blood pressure measurement. The arterial pressure of each rat was measured every week for 8 weeks and before each shRNA infusion. In total, 20 mean arterial pressure (MAP) and heart rate data points were collected over a period of 40 min between 8 a.m. and 11 a.m. each day until the end of this study, and the values were averaged.

### 2.6. Immunofluorescence and Immunohistochemistry

Immunofluorescence and immunohistochemistry staining were conducted using antibodies in accordance with the manufacturer’s instructions and previously described protocols. OTC-embedded brains were sectioned into several 14 μm-thick transverse sections located at the bregma coordinates −0.92 mm to −2.12 mm at −25 °C by a freezing microtome (CM1860, Leica, Wetzlar, Germany). The sections were then washed with PBS containing 0.1% Tween 20 3 times per 5 min, permeabilized with 0.3% Triton in Tris-buffered saline for 1 h at 37 °C, blocked using 5% goat serum with 0.2% Triton in Tris-buffered saline for 1 h, and incubated with a primary antibody in blocking buffer at 4 °C overnight. The following primary antibody was used for this analysis: TLR4 (1:300, ab22048, Abcam, Cambridge, UK). After washing 3 times for 5 min, the sections were incubated with secondary antibodies (Donkey Anti-Mouse IgG H&L (Alexa Fluor 488) (1:200, ab150105, Abcam). The cells were washed with PBS 3 times for 5 min each. After adding the antifade solution, immunofluorescence-stained sections were imaged using a fluorescence microscope (Nikon eclipse, 80i, Tokyo, Japan).

For 3,3-diaminobenzidine (DAB) staining, sections stained with primary antibodies targeting NKA α2 (1:300, ab222699, Abcam), MyD88(1:500, ab219413, Abcam), and Caspase-3 (Cleaved Caspase-3, 1:200, No. 9661, CST, Danvers, USA) were washed with PBS containing 0.1% Tween 20 for 1 h and then incubated with an anti-rabbit IgG (HRP) secondary antibody (1:200, ab7090, Abcam) in blocking buffer for 1 h. The HRP reaction was detected with a DAB kit (P0203, Beyotime, Shanghai, China) according to the manufacturer’s instructions. Processing was stopped with H_2_O, and sections were imaged using a microscope.

The superoxide anion level in the PVN was detected by Molecular Probes dihydroethidium (DHE). Brain sections (frozen, 14 μm thick) were incubated for 10 min with DHE (1 μmol/L, Sigma-Aldrich, Burlington, VT, USA) at 37 °C in the dark as previously described. The oxidative fluorescence intensity was detected at 585 nm by a fluorescence microscope imaging system (Nikon eclipse, 80i, Tokyo, Japan).

### 2.7. Western Blotting

Protein extracted from PVN tissues and the western blotting procedures were prepared as described previously [30]. Western blotting was used to measure protein expression. Tissues were homogenized in 100 μL of RIPA lysis buffer containing protease inhibitor cocktail. Protein was extracted from the homogenates, and the protein concentration in the lysate was measured using a BCA assay. The extracted protein (30 μg) was mixed with an equal volume of loading buffer, boiled for 5 min, and electrophoresed on 10–15% SDS-polyacrylamide gels. Then, the proteins were transferred onto polyvinylidene PVDF membranes. Nonspecific binding was blocked by incubating the membranes in 1% casein in phosphate-buffered saline-Tween 20 for 1 h at room temperature. The blots were then incubated overnight at 4 °C with the following primary antibodies: NKA α1 (dilution 1:400, sc-514661, Santa Cruz Biotechnology, Dallas, TX, USA), NKA α2 (dilution 1:500, ABP55363, Abbkine, CA, USA), NKA α3 (dilution 1:400, sc-71640, Santa Cruz Biotechnology), PKC γ (dilution 1:1000, ab71558, Abcam), p-Rac1 (dilution 1:300, ab5482 Abcam), T-Rac1 (dilution 1:500, ab129758, Abcam), TLR4 (dilution 1:1000, sc-293072, Santa Cruz), MyD88 (dilution 1:800, sc-74532, Santa Cruz Biotechnology), NF-κB p65 (dilution 1:500, sc-514451, Santa Cruz Biotechnology), and tumor necrosis factor α (TNF-α, dilution 1:500, sc-52746, Santa Cruz Biotechnology) in the PVN. After being washed with buffer four times for 10 min each time, the blots were incubated for 1 h with an HRP-conjugated secondary antibody (dilution: 1:10,000, Santa Cruz Biotechnology). The immunoreactive bands were visualized using enhanced chemiluminescence (Amersham ECL Plus, Cytiva, Marlborough, MA, USA). A β-actin antibody was used as an internal standard. The band densities were analyzed using Image J software.

### 2.8. Real-Time PCR

The procedure of real-time PCR has been described previously [31,32]. Briefly, rat brains were isolated and cut into coronal sections (−0.92 mm to −2.13 mm posterior to bregma). A block of hypothalamic tissue containing the PVN was excised from the coronal section. Total RNA was extracted from the microdissected PVN tissues using TRIzol reagent (No. 15596026, Invitrogen, Waltham, MA, USA) and reverse transcribed using oligo (dT) at 23 °C for 10 min, at 37 °C for 60 min, and at 95 °C for 5 min. The cDNA used was for real-time PCR with specific primers for NKA α1, NKA α2, NKA α3, and glyceraldehyde-phosphate dehydrogenase (GAPDH), which are shown in Table 1. The quantitative fold change in mRNA expression was calculated, and the data were normalized to the GAPDH mRNA level in each group.

### 2.9. Enzyme-Linked Immunosorbent Assay (ELISA) Results

We used ELISA kits to measure NKA activity (MBS7245054, MyBioSource, San Diego, CA, USA), ADP/ATP ratio (MAK135-1KT, Sigma-Aldrich, Burlington, VT, USA), NAD(P)H activity (ab186031, Abcam, Cambridge, UK), NF-κB activity (ABIN6958236, Antibodyies, Limerick, ME, USA), and SOD activity (S0101, Beyotime, Shanghai, China). For part II, we used Norepinephrine Assay Kit (H096, Nanjing Jiancheng Bioengineering Institute, Nanjing, China) to measure the NE levels in the plasma. The levels of oxidative stress-related indicators, including malondialdehyde (MDA, S0131, Beyotime), glutathione (GSH, S0073, Beyotime), oxidized glutathione (GSSH, S0053, Beyotime), and catalase (CAT, S0082, Beyotime) in the PVN, and pro- and anti-inflammatory cytokines, including IL-1β (PI303, Beyotime), MCP-1 (PC128, Beyotime), IL-6 (PI328, Beyotime), IL-10 (PI525, Beyotime), TNF-α (PT516, Beyotime), IL-8 (MBS9141543, MyBioSource), and IL-4 (BMS628, Thermo Fisher, Waltham, US) were also measured by ELISA kits (32).

### 2.10. ELISA for Ouabain-Like Compound (OLC)

For analysis of OLC levels in the PVN, we sent samples to the Department of Laboratory Medicine of the First Affiliated Hospital of Xi’an Jiaotong University [5,33,34,35]. The procedure is briefly described below. An anti-OLC antibody was raised in New Zealand white rabbits immunized with commercially available cardenolide ouabain conjugated to bovine serum albumin (BSA-OUA). Enzyme immunoassay plates were coated with ovalbumin-ouabain (OVA-OUA, 100 μL/well) at 4 °C overnight. In total, 50 μL of sample or ouabain standard (CAS. 11018896, Sigma-Aldrich, Burlington, VT, USA) was added to the appropriate well, and rabbit OLC antiserum (50 μL, dilution: 1:10,000) was added to each well. The plates were incubated at 37 °C for 2 h with continuous shaking and then rinsed four times for 5 min each time. A peroxidase-conjugated goat anti-rabbit IgG secondary antibody (No. AP132P, dilution 1:5000, Sigma-Aldrich) (100 μL) was added to each well, and the plates were incubated at 37 °C for 1 h with continuous shaking and then rinsed four times for five minutes each time. The presence of peroxidase enzyme in each well was assessed by the addition of 100 μL of 3,3-5,5-tetramethylbenzidine base (TMB) substrate solution (No. T0440, Sigma-Aldrich). After 30 min, the reaction was terminated by the addition of H_2_SO_4_ (50 μL). The absorbance of each well was measured at 450 nm using a microplate reader (Well Scan MK3, Thermo Scientific, Waltham, MA, USA). The concentration of OLC in each sample was calculated from the absorbance value according to the ouabain standard curve [36,37,38,39].

### 2.11. Statistical Analysis

The data are presented as the mean ± SEM. Statistical analyses were performed using GraphPad Prism software version 8.0. Blood pressure and MAP data were analyzed by repeated measures ANOVA. One-way ANOVA followed by Tukey’s posthoc test was used to determine the significance of differences in Western blotting and ELISA data.

## 3. Results and Statistical Analyses

### 3.1. Effect of High Salt Diet on NKA Subunits, Na^+^/K^+^-ATPase, Oxidative Stress, and Inflammation in the PVN

Western blotting showed that compared with a normal salt diet, a high salt diet increased the PVN expression of NKA α1 and NKA α2. However, the increase of NKA α3 expression in the PVN was not significantly different from the normal salt diet (*p* > 0.05) (Figure 1B,C). Real-time PCR revealed that the high salt diet significantly increased the PVN mRNA levels of NKA α1 and NKA α2 (*p* < 0.05) but not the mRNA level of NKA α3 (*p* > 0.05) compared to the normal salt diet group (Figure 1D). The protein of NKA α3 did not change between the two groups, which is consistent with the real-time PCR results. In addition, we also measured NKA activity, ADP/ATP ratio, NAD(P)H activity, NF-κB activity, and OLC level to explore the effect of long-term diet on the NKA activity, oxidative stress, and inflammation in the PVN. The results showed that a high salt diet decreased the NKA activity and ADP/ATP ratio (*p* < 0.05, Figure 1E,F), but increased the NAD(P)H activity, NF-κB activity, and OLC level in the PVN (*p* < 0.05, Figure 1G–I) compared to the normal salt group, which means the high salt diet had a significant effect on the NKA, oxidative stress, and inflammation in the PVN.

### 3.2. Effect of Decreased NKA α2 in the PVN on Mean Arterial Blood Pressure, Sympathetic Activity, NKA, and ADP/ATP Ratio in Salt-Induced Hypertension

Based on the first part of the study, we designed NKA α2 knockdown short hairpin RNA (shRNA) which was carried in the adenovirus-associated virus (AAV). The MAP was decreased in the group of salt-induced hypertensive rats that received bilateral microinjection of NKA α2 shRNA into the PVN for 8 weeks compared to the NKA α2 scramble group with high salt diet (Figure 2B, *p* < 0.05). The level of NE (a substance that is released predominantly from the ends of sympathetic nerve fibers) in the plasma was lower in the group of hypertensive rats that received microinjection of NKA α2 shRNA into the PVN than in the control group (Figure 2C, *p* < 0.05). Moreover, NKA activity and the ADP/ATP ratio were higher but OLC level was lower in the HS+ NKA α2 shRNA group than in the hypertensive group (Figure 2D–F, *p* < 0.05).

### 3.3. Effect of Decreased NKA α2 in the PVN on the Level of NKA α2, PKC γ, and p-Rac1 in Salt-induced Hypertension

**Figure 3:**
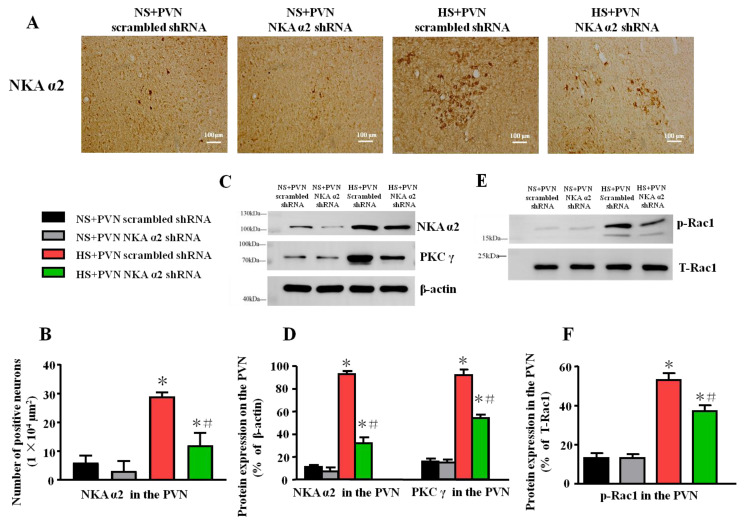
Effect of decreased NKA α2 in the PVN on the level of NKA α2, PKC γ, and p-Rac1 in salt-induced hypertension. After microinjection of NKA α2 shRNA in the PVN, the protein expression of NKA α2, Protein Kinase Cγ (PKC γ) and phosphorylated Ras-related C3 botulinum toxin 1 (p-Rac1) in the PVN in different groups. (**A**) Immunohistochemistry for NKA α2 in the PVN in different groups. (**B**) Column diagram on the number of NKA α2 positive neurons in the PVN in different groups. (**C**) A representative immunoblot of protein expression and (**D**) densitometric analysis of NKA α2, PKC γ, and p-Rac1 in the PVN in different groups. (**E**) A representative immunoblot and (**F**) densitometric analysis of p-Rac1 in the PVN in different groups. * *p <* 0.05 versus the normal salt groups (the normal salt + NKA α2 shRNA or normal salt + scrambled shRNA group); # *p <* 0.05, the high salt + NKA α2 shRNA group versus the high salt + scrambled shRNA group. Values are expressed as the mean ± SEM (*n* = 6).

After microinjection of NKA α2 shRNA in the PVN, we measured the level of expression of NKA α2 via immunohistochemistry. The result shows that the number of NKA α2 positive neurons after microinjection of NKA α2 shRNA in hypertensive rats is lower than the PVN scrambled shRNA in the PVN (*p* < 0.05, Figure 3A,B). Thus, it indicates that NKA α2 shRNA suppresses the expression of NKA α2 in the PVN.

Because PKC γ/Rac1/NAD(P)H is a close signal pathway regarding oxidative stress in the PVN in salt-induced hypertension, the high salt diet increases the expression of NKA α2, PKC γ, and p-Rac1 in the PVN compared to vehicle. After microinjection of NKA α2 shRNA in the PVN, the expression of NKA α2 was lower in this group relative to the group with microinjection of scrambled shRNA in the PVN (*p* < 0.05). Meanwhile, the expression of PKC γ in the HS+PVN NKA α2 shRNA was lower than the HS + PVN scrambled shRNA (*p* < 0.5) (Figure 3C,D). In addition, the p-Rac1 protein expression was lower in the HS + PVN NKA α2 shRNA than in the HS + PVN scrambled shRNA (*p* < 0.05) (Figure 3E,F).

### 3.4. Effect of Decreased NKA α2 Expression in the PVN on Oxidative Stress in Salt-Induced Hypertension

**Figure 4:**
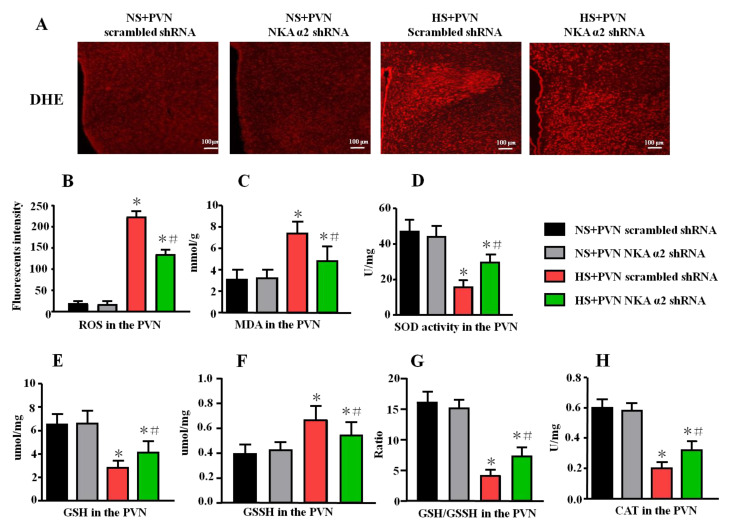
Effect of decreased NKA α2 expression in the PVN on the oxidative stress in salt-induced hypertension. Rac1 is one of the important NAD(P)H subunits, which contributes to oxidative reactive oxygen species (ROS) production. DHE can detect the superoxide anion levels in the tissues. (**A**) Immunofluorescence for DHE detecting. (**B**) Column diagram showing the immunofluorescent intensity of DHE in the PVN in different groups. ELISA results showing the levels of MDA, SOD, GSH, GSSH, GSH/GSSH, and CAT in the PVN. (**C**) The level of MDA, (**D**) SOD activity, (**E**) GSH, (**F**) GSSH, (**G**) GSH/GSSH ratio, and (**H**) CAT activity in the PVN in the different groups. * *p <* 0.05 versus the normal salt groups (the normal salt + NKA α2 shRNA or normal salt + scrambled shRNA group); # *p <* 0.05, the high salt + NKA α2 shRNA group versus the high salt + scrambled shRNA group. Values are expressed as the mean ± SEM (*n* = 6).

In addition, the fluorescent-labeled dihydroethidium (DHE) staining can detect the superoxide anion levels in the tissues. Our results showed that the fluorescent intensity in the HS + PVN NKA α2 shRNA is lower than the HS + PVN scrambled shRNA (*p* < 0.05, Figure 4A,B), which indicated that microinjection of NKA α2 shRNA in the PVN in hypertensive rats decreases ROS overproduction in the PVN.

The level of malondialdehyde (MDA), glutathione (GSH), oxidized glutathione (GSSH), GSH/GSSH ratio, catalase (CAT), and superoxide dismutase (SOD) activity in the PVN were measured using ELISA. A high salt diet increased the level of MDA and GSSH but decreased the levels of GSH, GSH/GSSH ratio, CAT, and SOD activity (*p* < 0.05). After microinjection of NKA α2 shRNA into the PVN of hypertensive rats, the level of MDA and GSSH were lower (Figure 4B, *p* < 0.05). However, the levels of SOD, GSH, GSH/GSSH ratio, and CAT were higher in the NKA α2 shRNA-microinjected group than them in the hypertensive group (Figure 4C–E, *p* < 0.05).

### 3.5. Effect of Decreased NKA α2 Expression in the PVN on the Expression of TLR4 and MyD88 in Rats with Salt-Induced Hypertension

**Figure 5:**
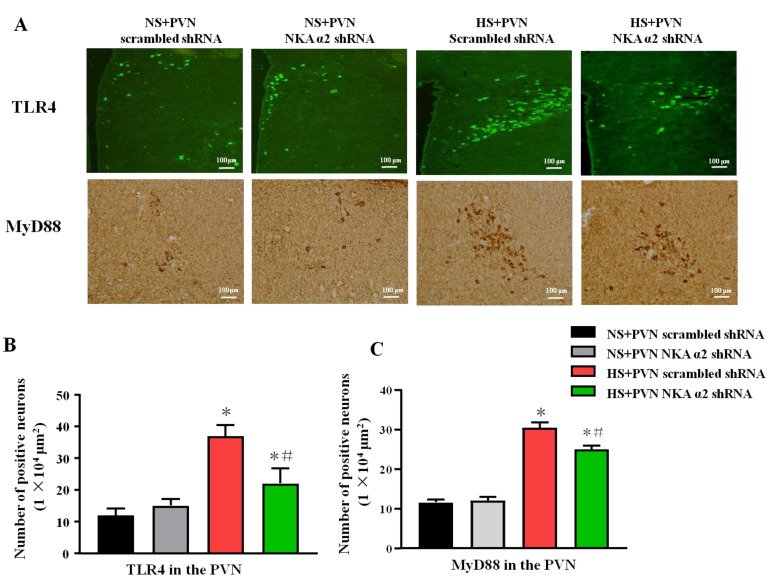
Effect of decreased NKA α2 expression in the PVN on the expression of TLR4, NF-κB p65 in rats with salt-induced hypertension. After microinjection of NKA α2 shRNA in the PVN, we measured the protein expression of TLR4 and MyD88 via immunohistochemistry and immunofluorescence. (**A**) Immunofluorescence for TLR4 and immunohistochemistry for MyD88 in the PVN in different groups. (**B**,**C**) Column diagram showing the effects of NKA α2 shRNA microinjections on the number of TLR4 and MyD88 positive neurons in the PVN in different groups. * *p <* 0.05 versus the normal salt groups (the normal salt + NKA α2 shRNA or normal salt + scrambled shRNA group); # *p <* 0.05, the high salt + NKA α2 shRNA group versus the high salt + scrambled shRNA group. Values are expressed as the mean ± SEM (*n* = 6).

The signaling pathway of TLR4/MyD88/NF-κB contributes to the inflammation. The immunohistochemistry staining results showed that the high salt diet increases the number of TLR4 and MyD88 positive neurons in the PVN. Bilateral PVN microinjection of NKA α2 shRNA in hypertensive rats decreases the PVN positive neurons of TLR4 and MyD88 (*p* < 0.05) (Figure 5A–C).

### 3.6. Effect of Decreased NKA α2 Expression in the PVN on the Expression of TLR4, MyD88, NF-κB p65, TNF-α, and Caspase-3 in Rats with Salt-Induced Hypertension

**Figure 6:**
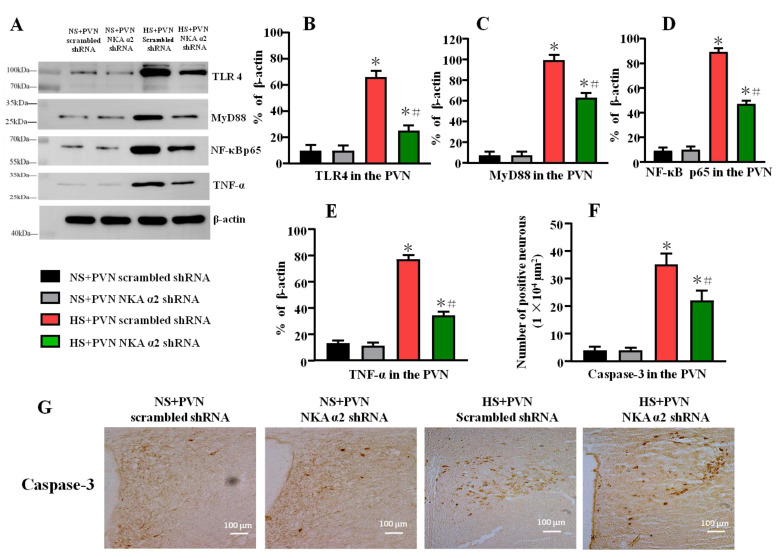
Effect of decreased NKA α2 expression in the PVN on the expression of TLR4, MyD88, NF-κB p65, and TNF-α in rats with salt-induced hypertension. (**A**) A representative immunoblot densitometric analysis of protein expression of TLR4, MyD88, NF-κB p65, and TNF-α in the PVN in different groups. (**B**–**E**) Column diagram showing the effects of microinjections NKA α2 shRNA in the PVN on the protein expression of TLR4, MyD88, NF-κB p65, and TNF-α. (**F**,**G**) Immunohistochemistry and column diagram showing the effects of NKA α2 shRNA microinjections on the number of Caspase-3 positive neurons in the PVN in different groups. * *p <* 0.05 versus the normal salt groups (the normal salt + NKA α2 shRNA or normal salt + scrambled shRNA group); # *p <* 0.05, the high salt + NKA α2 shRNA group versus the high salt + scrambled shRNA group. Values are expressed as the mean ± SEM (*n* = 6).

Long-term salt intake increased the protein levels of TLR4, MyD88, NF-κB p65, and TNF-α (*p* < 0.05) in the PVN. After decreasing the level of NKA α2 in the PVN in salt-induced hypertension, the TLR4, MyD88, NF-κB p65, and TNF-α protein in the PVN were lower than the control hypertensive rats (Figure 6A–E, *p* < 0.05).

High salt diet increases the number of Caspase-3 positive neurons in the PVN. Bilateral PVN microinjection of NKA α2 shRNA in hypertensive rats decreases the PVN positive neurons of Caspase-3 (Figure 6F,G, *p* < 0.05).

### 3.7. Effect of Decreased NKA α2 Expression in the PVN on Cytokine Levels in Rats with Salt-Induced Hypertension

**Figure 7:**
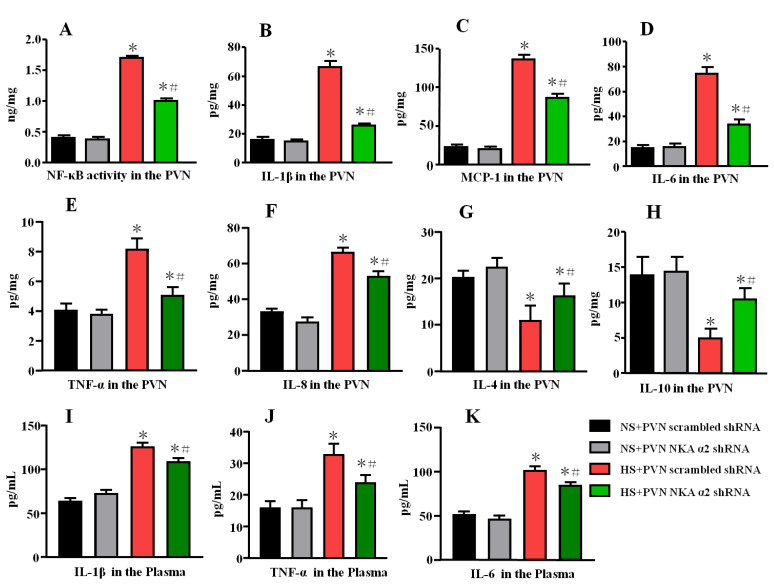
Effect of decreased NKA α2 expression in the PVN on cytokine levels in rats with salt-induced hypertension. (**A**) NF-κB activity in the PVN. (**B**) The PVN level of interleukin 1β (IL-1β). (**C**) The PVN level of Monocytechemoattractantprotein-1 (MCP-1). (**D**) The PVN level of IL-6. (**E**) The PVN level of TNF-α. (**F**) The PVN level of IL-8. (**G**) The PVN level of IL-4. (**H**) The PVN level of IL-10. (**I**) The plasma level of IL-1β. (**J**) The plasma level of TNF-α. (**K**) The plasma level of IL-6 in the different groups. * *p <* 0.05 versus the normal salt groups (the normal salt + NKA α2 shRNA or normal salt + scrambled shRNA group); # *p <* 0.05, the high salt + NKA α2 shRNA group versus the high salt + scrambled shRNA group. Values are expressed as the mean ± SEM (*n* = 6).

A high salt diet increased NF-κB activity and the levels of the IL-1β, MCP-1, and IL-6, TNF-α, IL-8 but decreased the level of the anti-inflammatory cytokines IL-4 and IL-10 (*p* < 0.05) in the PVN. NF-κB activity and the levels of the IL-1β, MCP-1, and IL-6, TNF-α, IL-8 were decreased but the expression of the anti-inflammatory cytokines IL-4 and IL-10 in the PVN was increased in the group of hypertensive rats that received microinjection of NKA α2 shRNA into the PVN compared to the control group (Figure 7A–H, *p* < 0.05).

Meanwhile, we also measured the plasma levels of IL-1β, TNF-α, and IL-6 by ELISA. High salt diet increases the plasma levels of IL-1β, TNF-α, and IL-6. Bilateral PVN microinjection of NKA α2 shRNA in hypertensive rats decreases the plasma levels of IL-1β, TNF-α, and IL-6 (Figure 7I–K, *p* < 0.05).

## 4. Discussion

This study investigated the effect of NKA α2 in the PVN and its specific mechanism on oxidative stress and inflammation in salt-induced hypertension. The data from this study suggested that, first, a high salt diet increased NKA α2 expression, oxidative stress, inflammation, and OLC level, but suppressed Na^+^/K^+^-ATPase activity and the ADP/ATP ratio in the PVN. Second, decreased NKA α2 expression not only improved NKA activity and ADP/ATP ratio but also suppressed the PKC γ/Rac1/NAD(P)H and TLR4/MyD88/NF-κB signaling pathways in the PVN, thus attenuating sympathetic nerve activity and MAP in salt-induced hypertension. This study found that NKA α2 in the PVN elicits PKC γ/Rac1/NAD (P)H-dependent oxidative stress and TLR4/MyD88/NF-κB-induced inflammation in the PVN and augments sympathetic activity during the development of salt-induced hypertension.

Blaustein reported that long-term salt intake increases the level of NaCl in the CSF and elevates OLC secretion in the brain, which can inhibit NKA activity in the brain [1], which is consistent with the OLC results in this study. Thus, long-term salt diet can regulate NKA activity in the brain, which contributes to the downstream sympathetic outflow. Notably, NKA comprises α subunits, β subunits, and other subunits. In addition, NKA α subunits probably can bind to cardiac glycosides and are responsible for the ion transport and catalytic properties of these enzymes. Based on previous studies, NKA α1, NKA α2, and NKA α3 were distributed in the central neural system [19]. Thus, we mainly focused on NKA α1, NKA α2, and NKA α3 in the PVN of salt-induced hypertensive rats. To explore the mechanism of NKA, the protein and mRNA level of three subunits in the PVN in the high salt group were measured first. It caused the PVN levels of NKA α1 and NKA α2 to be higher in the hypertensive group than in the control group (Figure 1B–D). The change in NKA α2 expression was much greater than that in NKA α1 expression after a long-term high salt diet. Thus, we concluded that a high salt diet has a significant effect on NKA α2 that is probably the target for subsequent hypertension responses. Meanwhile, the high salt diet suppressed NKA activity but elicited oxidative stress and inflammation in the PVN, which is consistent with our previous studies [40,41]. In addition, Srikanthan et al. (2016) found that the Na/K-ATPase/ROS amplification loop has a significant effect on oxidative stress related to cardiovascular diseases such as hypertension and heart failure [8]. Leite’s study also showed that the NKA α2 isoform mediates LPS-induced neuroinflammation [42]. Thus, we speculated that suppressing NKA activity may activate downstream oxidative stress and inflammatory signaling pathways in the PVN and promote hypertension.

In the second part of our study, after we added NKA α2 shRNA to the PVN in salt-induced hypertensive rats, blood pressure and sympathetic activity decreased, which means that NKA α2, as an important target, in the PVN regulated the hypertensive responses. Furthermore, our results also showed that decreasing NKA α2 in the PVN increased NKA activity, but decreased the level of OLC, NAD(P)Hase activity, and NF-κB activity. Huysse (2009) also showed that NKA α2 may be a target of OLC in the brain when the CSF Na^+^ concentration is increased in salt-dependent hypertension [19]. Thus, we concluded that the high salt diet inhibited NKA pump activity probably by OLC and led to ion transport failure. Then, an imbalance in intracellular and extracellular Na^+^ and K^+^ distribution in the PVN provoked the oxidative stress and inflammation so as to increase blood pressure and sympathetic activity.

Several reports have indicated that inhibited NKA decreases the activity of antioxidant enzymes, including SOD and CAT, alters oxidative stress parameters, and promotes overproduction of ROS in the brain [43,44,45]. Our previous study showed that PKC γ upregulates the expression of Rac1, which is an essential subunit for NAD(P)H oxidase activation and the production of superoxide in the context of salt-induced hypertension [26]. Therefore, we measured PKC γ and p-Rac1 expression in the PVN to explore the specific mechanism by which NKA α2 regulates oxidative stress. Microinjection of NKA α2 shRNA into the PVN reduced PKC γ and p-Rac1 expression, NAD(P)H enzyme activity, and sympathetic activity, but increased antioxidant production in the PVN in hypertensive rats. Therefore, we concluded that high salt intake probably provokes PKC γ/Rac1/NAD(P)H pathway-dependent oxidative stress by regulating NKA α2 levels in the PVN during salt-induced hypertension development.

In addition, Jiang et al. (2018) reported that a high salt diet augments sympathetic nerve activity and arterial blood pressure by increasing the PIC levels in the PVN [46]. The NKA pump also regulates the neuroinflammatory responses in microglia cells [11], which are important for modulating neuroinflammatory responses in the brain. Several studies have reported that toll-like receptors (TLRs), specifically TLR4, modulate the inflammatory signaling pathway [47,48]. Our previous results confirmed that the TLR4/MyD88/NF-κB signaling pathway in the PVN regulates the downstream transcription factors of cytokines in the context of hypertension [27]. We also showed that high salt intake suppressed NKA activity in the PVN, and upregulated TLR4, MyD88, and NF-κB p65 expression so as to decrease the levels of PICs in the PVN. However, bilateral microinjection of NKA α2 shRNA into the PVN reversed changes in the TLR4-dependent pathway and PICs expression and decreased sympathetic activity and blood pressure. Hence, NKAα2 regulates the TLR4/MyD88/NF-κB signaling pathway and induces the PVN neuroinflammatory responses in the PVN, thus elevating blood pressure and sympathetic activity in the context of salt-induced hypertension.

**Figure 8:**
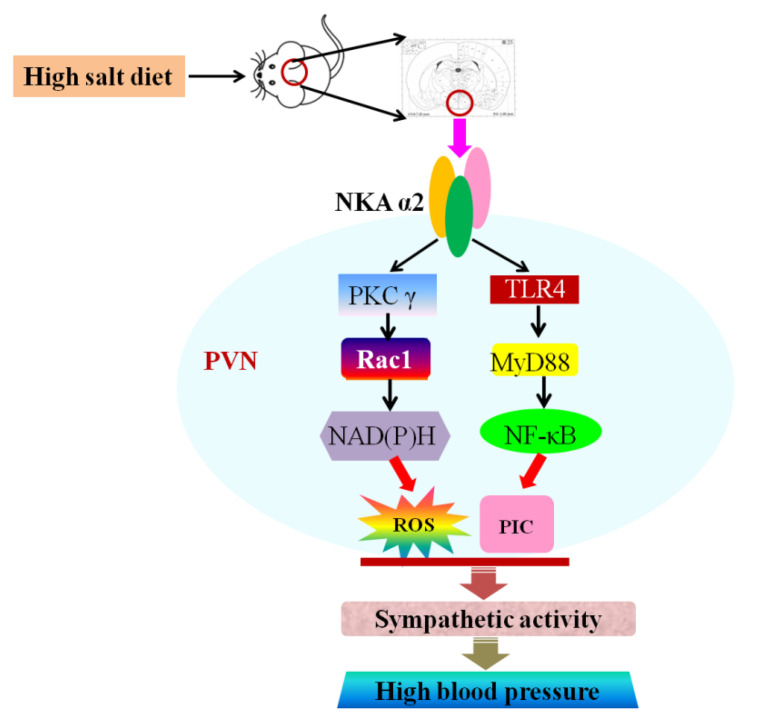
Graphical abstract. The proposed mechanism underlying the effect of NKA α2 in the PVN. NKA α2 in the PVN elicits PKC γ/Rac1/NAD (P)H-dependent oxidative stress and TLR4/MyD88/NF-κB-induced inflammation in the PVN, which augments the MAP and sympathetic activity during the development of salt-induced hypertension.

In conclusion, an excessive salt diet increased NKA α2 expression, ROS level, cytokines expression, and OLC level, but suppressed Na^+^/K^+^-ATPase activity and the ADP/ATP ratio in the PVN. NKA α2 in the PVN elicits PKC γ/Rac1/NAD (P)H-dependent oxidative stress and TLR4/MyD88/NF-κB-induced inflammation in the PVN, thus increasing the MAP and sympathetic activity during the development of salt-induced hypertension (Figure 8).

## 5. Limitations

Some questions in this study still need to be further explored and discussed. Our study mainly focused on physiological responses. Bilateral PVN microinjection of knockdown NKA α2 AAV was administered to high salt-induced hypertensive rats and then PVN levels of PCK γ-related oxidative stress proteins and TLR4-related inflammation proteins were measured. Considering our previous studies and others, we deduced that NKA α2 in the PVN elicits PKC γ/Rac1/NAD (P)H-dependent oxidative stress and TLR4/MyD88/NF-κB-induced inflammation in the PVN in salt-induced hypertension. However, we also need some more research to explore the direct evidence of NKA α2 regulation of their downstream pathways. For example, PKC γ or TLR4 genes knockdown or microinjection of related inhibitors into the PVN should be performed in vitro and/or in vivo, which can directly prove that NKA α2 regulates Rac1-dependent oxidative stress and TLR4-induced inflammation in the PVN during the development of high salt-induced hypertension. However, that is another study related to numerous experiments and analyzed data, which cannot be presented in this paper in its entirety. Therefore, future research needs to explore direct evidence of NKA α2 and its signaling pathways.

## 6. Conclusions

Numerous studies have reported that Na^+^/K^+^-ATPase (NKA) plays a vital role on regulation of cardiovascular function. The results of this study suggested that the change in NKA α2 isoform expression was much greater than that in NKA α1 and NKA α3 expression after a long-term high salt diet. Meanwhile, the high salt diet suppressed NKA activity and elicited oxidative stress and inflammation in the PVN. After bilateral PVN microinjection of NKA α2 shRNA, the blood pressure and sympathetic activity have a significant decrease. Additionally, decreasing NKA α2 not only reduced the level of OLC, NAD(P)Hase activity, but also suppressed the PKC γ and p-Rac1 expression in the PVN. Furthermore, NKA α2 shRNA restored the balance of pro- and anti- inflammatory cytokines as well as decreased the TLR4, MyD88, and NF-κB p65 expression in the PVN during the development of hypertension. Overall, NKA α2 in the PVN probably as a target regulates the cardiovascular responses and elicits PKC γ/Rac1/NAD (P)H-dependent oxidative stress and TLR4/MyD88/NF-κB-induced inflammation in the PVN, thus increasing MAP and sympathetic activity during the development of salt-induced hypertension.

However, further in-depth studies are needed to determine the direct evidence of NKA α2 regulation of their downstream pathways that regulates Rac1-dependent oxidative stress and TLR4-induced inflammation in the PVN during the development of high salt-induced hypertension.

## Figures and Tables

**Table 1:** Primer sequences designed for qPCR in this study.

Name	Forward	Reverse
**NKA α1**	TTGTGGCTCAGTAAAGGACA	CCAATGAGGGTGAGAGCAA
**NKA α2**	GAGACACTGCAGGAGATG	TGAGGAATCACCCACAGG
**NKA α3**	ATCCTGAAGAGGGACGTG	GTGCATGAGACAGAAGACTC
**GAPDH**	ATGGAGAAGGCTGGGGCTCACCT	AGCCCTTCCACGATGCCAAAGTTGT

## Data Availability

Data is contained within the article.
